# Supplementary material for: Are carbon emissions trading and green financial instruments synergistic? -Comprehensive quantitative research based on content analysis
Source: PLoS One. 2024 Mar 7;19(3):e0298601. doi: 10.1371/journal.pone.0298601 (PMC10919601; doi:10.1371/journal.pone.0298601)
Supplement: S1 Data — (ZIP) [file pone.0298601.s001.zip › Data/地级市Z_F工作报告.zip!/┴╔─■/░░╔╜╩╨/└┤╫╘╣1⁄2╓┌║┼í╛┬φ┐╦╩2╛▌═°í┐_2010.docx]

各位代表：

现在，我代表市人民政府向大会报告工作，请予审议，并请市政协各位委员提出意见。

一、2009年政府工作回顾

2009年是我市战胜金融危机严重影响，实现经济社会大发展、快发展的一年。在市委领导下，在市人大、市政协监督支持下，市政府深入学习实践科学发展观，按照“围绕一个核心、加速两个融入、推进三大任务”的总体要求，只争朝夕，努力工作，较好地完成了市十四届人大二次会议确定的各项任务。

预计全年实现地区生产总值1915亿元，比上年增长18.5%；地方财政一般预算收入123亿元，按可比口径增长33.7%；全社会固定资产投资1050亿元，增长50%；规模以上工业增加值735亿元，增长17.5%；实际利用外商直接投资7.38亿美元，增长27%；引进内资530亿元，增长30%；社会消费品零售总额460亿元，增长24.9%；城市居民人均可支配收入16580元，农民人均纯收入8530元，分别增长10%和17%。

过去一年，我们主要做了七个方面工作：

（一）积极应对金融危机影响，全力以赴扩内需保增长

面对金融危机给钢铁和矿产品市场带来的严重冲击，我们变压力为动力，化危为机，积极采取措施保增长。

争取资金保增长。利用国家积极的财政政策，努力争取国债资金，291个项目到位资金6.2亿元。利用国家适度宽松的货币政策，积极推进银企对接，帮助企业协调银行贷款300亿元，全市银行业金融机构贷款余额为967亿元，增长35.5%。利用国家鼓励发展民间金融政策，大力发展县域金融业，发展村镇银行6家、小额贷款公司35家。

抓住机遇保增长。抓住国家加大对基础设施、社会事业设施、改善民生的投入和鼓励房地产发展的机遇，大力支持企业发展铁路用轨、建筑钢材、工程机械等产品，大力发展保障性住房和工业地产、商业地产、住宅地产，大力建设教育、文化、医疗等社会设施。

政策扶持保增长。为支持企业发展，及时果断出台了一系列政策措施。安排2亿元，给予鞍钢等钢铁企业及镁制品企业贷款贴息和资金支持；安排3000万元，鼓励企业扩大外贸出口；发挥担保公司作用，为全市中小企业解决发展资金18亿元；安排1亿元，支持企业调整产品结构、科技创新和节能降耗；安排3亿元，支持设施农业发展；安排1000万元，支持服务业发展。

加大投资保增长。全年完成工业投资550亿元，增长35.5%。完成服务业投资257亿元，增长2.1倍，其中房地产投资181亿元，增长1.7倍。完成农业投资51亿元，增长3.4倍。完成基础设施投资116亿元，增长2倍。完成社会设施投资76亿元，增长9.8倍。

招商引资保增长。全面吸引央企、境外、南方民营和本地资本投资鞍山。全市开工建设项目1251个，总投资1511亿元。在全面引进产业资本的同时，大力引进金融资本，浦发、广发、民生、华夏、招商、光大6家股份制银行，中意、阳光人寿2家保险公司及大通证券先后进驻。现在的鞍山已经成为产业资本高速增长、金融资本快速集聚的城市。

过去一年，是鞍山战胜金融危机逆势而上、脱颖而出的一年。全市上下充分展示了在困难重重中迎难而上、锐意进取的昂扬斗志，表现出不达目标誓不罢休的工作锐气。实践证明，没有什么困难能够阻挡我们振兴鞍山的前进步伐！

（二）以大开放大开发促进大发展，全面构建鞍山发展新格局

“两个融入”格局已经形成。以打造辽宁沿海经济带和沈阳经济区互动发展区为目标，以鞍海经济带建设为重点，全面推进两个融入。公路全面融入，腾达通海产业大道和沈西工业走廊通海产业大道鞍山段加快建设，海岫高速公路全线开工，鞍山至台安高速公路完成规划设计。铁路全面融入，哈大快客、沈阳经鞍山至北京快客、岫庄铁路全面建设，沈辽鞍营城际轨道交通鞍海段完成预可研论证和设计。航运全面融入，完成鞍山机场改造，力争春节前开通直飞北京航线。

城市和县域空间格局已经形成。以规划为先导，拓展发展空间，优化城乡布局。城市空间格局全面拉开。完成城市总体规划修编和土地利用规划调整，以基础设施建设为先行，积极扩大城市空间，城市开发面积由154平方公里向624平方公里全面拓展。形成了以东部东环路、南部鞍南大道、西部腾达大道、北部灵山路为环线，以盛达大道、四达大道、建国路、汇园路等为南北干线，以南环路、千山路、万水河北路等为东西干线，工业区、商业区、居住区合理布局的城市骨架。工业空间全面拓展，沿腾达大道，全面建设了达道湾、灵山、高新区（西区）、宁远、汤岗子农高区等五大工业园区。服务业空间全面拓展，打造了四达现代服务业集中区等五大服务业集中区和千山路、建国路等精品商业带。县域空间格局全面形成。海城以海西新区建设为重点，沿海城河把老城区和西柳镇连为一体，形成大海城空间格局；台安、岫岩加速县城改造，形成了行政区、生活区和产业区。海城开发区、腾鳌开发区、台安九股河工业区、岫岩雅河工业区等县域工业园区不断发展。鞍羊线、鞍营线、张庄线、腾南线设施农业示范带加速发展，海城耿庄、台安新开、岫岩前营农高区全面建设。

产业发展格局已经形成。发挥比较优势，培育和发展支柱产业。全市重点发展精特钢和钢铁深加工、装备制造及工业自动化、镁制品等矿产品深加工、化工新材料、光电等五大主导产业集群。海城工业重点发展以镁合金、镁化工、镁建材、镁铸造为代表的镁制品深加工产业集群，积极培育化工塑料产业集群，农业重点发展大蒜、生姜、辣根、圆葱等“四辣”农产品种植及深加工；台安工业重点发展木业产业集群，积极培育农药产业集群，农业重点发展鸭鹅等家禽养殖及深加工；岫岩工业重点发展特石深加工产业集群，积极培育以中草药深加工为代表的保健品产业集群，农业重点发展食用菌及深加工。

鞍山发展新格局的形成，促进了全市经济有序和可持续发展。工业向园区集中，服务业向集中区和城区集中，设施农业向示范带集中，正在实现集中式布局、集群化发展和集约化生产。

（三）积极推进结构调整，大力转变发展方式

工业结构调整取得新进展。全面发展五大支柱产业。精特钢和钢铁深加工产业集群做大做强，重点发展了鞍钢30万吨取向硅钢、东北特钢5万吨不锈钢极薄带和2万吨不锈钢焊管、鞍钢重机45万吨精品铸钢、辽宁新富与北京太行矿业合资80万吨不锈钢和永安10万吨马口铁等精特钢项目，推进了峰驰公司50万吨冷轧超薄板等板材深加工、鞍钢60万吨精品线材和后英80万吨高档线材等线材深加工、香港恒江50万吨无缝钢管等管材深加工、宝得150万吨H型钢等钢结构项目，钢材深加工率由35%提高到42%。装备制造及工业自动化产业集群发展壮大，重点推进了鞍钢重机大型铸锻基地、中铁物资与紫竹集团合资生产铁路组件等装备制造项目，荣信股份自动化成套系统、宏源公司与西门子合作5000套智能网络中低压控制柜等工业自动化项目，装备制造占全市工业比重由12%提高到17%。化工新材料产业集群异军突起，重点发展了鞍钢60万吨煤焦油、中橡化工16万吨碳黑、中钢集团16万吨针状焦和核石墨、中化国际4000吨有机颜料等煤焦油精深加工项目，鞍钢15万吨苯加氢等苯系深加工项目，国锐化工尼龙66盐、广东雅佳5万吨树脂涂料等石化深加工项目，正在形成销售收入超千亿元的产业集群。矿产品深加工产业集群迅猛发展，重点发展了晋德矿业1万吨金属镁板材等镁合金、海城精华矿业2万吨氢氧化镁无机阻燃剂等镁化工、后英集团100万立方米玻镁板等镁建材、中国镁业30万套汽车配件等镁铸造项目，矿产品深加工率由47%提高到55%。光电产业集群快速发展，重点招商推进了兵装天威集团100兆瓦多晶硅和150兆瓦双节薄膜电池等新能源、九夷三普镍氢电池等新动力、香港伟志LED等新光源、鞍山双桥公司地源热泵等新热源项目。全年开工建设工业项目1000多个，新增规模以上企业432家，总数达到2400家。鞍山工业正在由钢铁一柱擎天向五大支柱产业并举发展转变。

现代农业实现快速发展。大力发展设施种植业、设施养殖业、设施渔业和设施林业，新发展设施农业20万亩，新增标准化温室大棚4.9万栋、标准化养殖小区440个，全市设施农业面积发展到93万亩，占农业比重达到25%。全面推行农业保险，增强了农业抵抗自然灾害能力。一县一品特色农业主导产业集群加速发展，海城“四辣”农产品种植面积达到3万亩，台安鸭鹅等家禽养殖规模达到6000万只，岫岩食用菌培植规模达到8000万盘。农产品深加工产业迅猛发展，以农产品深加工园区和龙头企业为重点，新上农产品深加工项目126个，规模以上农业产业化龙头企业发展到400家。全年农业实现增加值86亿元，增长11%。鞍山正在由传统农业向现代农业转变。

现代服务业实现大发展。服务业集中区全面建设，四达现代服务业集中区已有东北MALL、红星美凯龙、凯兴物流、鞍山图书城等一批大市场开工建设；中国鞍山钢铁大市场已有中船重工200万吨船用板和鞍钢300万吨板材深加工配送中心、北方钢铁交易中心等项目开工建设；鞍辽路五金机电大市场的工程机械配件城基本建成，机电五金交易大厦开工建设；解放北路汽车贸易产业带汽车4S店发展到40家，瑞盛国际汽配城即将竣工；海城西柳中国商贸城建成并即将开业，商贸物流园投入使用。精品商业带加速发展，重点打造千山路、建国路、胜利路等精品商业带，家乐福、大商新玛特、华润万家、凯兴冷鲜品市场等已经建成，乐都汇广场、兴隆大家庭、新兴商贸城、大润发铁东店、新百盛广场等一大批精品商业项目开工建设。服务业态全面提升，全市有2360户个体工商户转型升级为法人单位，大力推进法人进商场，实现由档口式经营向统一经营转变。全年新上服务业项目227个，新增规模以上商业企业180家，总数达到980家；实现第三产业增加值800亿元，增长24%；实现旅游总收入140亿元，增长25%。服务业成为推动全市经济快速增长的重要力量，成为城区经济发展的重要支撑。

可持续发展能力不断增强。节能减排取得新成效，全年实施重点节能技术改造项目134个；依法关停“五小”企业150家；通过发展集中供热，逐步取缔城区20吨以下燃煤锅炉；对全市电厂、锅炉和烧结机实施脱硫改造工程；判甲炉等7个污水处理厂开工建设，全市污水处理能力达到46万吨。科技创新取得新进展，成功收购10家国外科技型企业，引进14个海外研发团队；发展企业技术中心、工程研究中心和实验室71家；国家级高新技术企业发展到53家。鞍山被评为全国科技进步先进市。

鞍钢围绕“全面腾飞”目标，积极开展与国外大企业合资合作，加速推进跨区域重组，大力发展精特钢和非钢产业，国际化经营、多角化发展迈出重大步伐；积极应对金融危机带来的严重影响，全力以赴保生产、保出口、保城市供气，为全市保增长、调结构、转方式、重民生作出了重大贡献。

通过结构调整，地方经济高速增长，规模以上工业增加值增长40%；三次产业协调发展，第三产业增加值比重提高到42%，税收比重提高到47.6%；单位生产总值能耗下降6%，主要污染物排放量减少5%。

（四）加大支持力度，促进县域经济大发展快发展

2009年是县域经济快速发展的一年。全年县域实现地区生产总值936.2亿元，增长29.4%；一般预算收入完成39亿元，增长48.9%，在全省名列前茅。继续加大了对县域经济支持力度，全年投入县域发展资金13.5亿元，连续四年增长50%。其中，支持三县（市）各2亿元，用于县域园区基础设施建设；支持三县（市）各1亿元，用于发展设施农业。县域工业实现大发展，围绕园区、集群、企业和项目，大力推进企业创新改造和招商引资上项目。投资工业项目477个，总投资240亿元，新增规模以上工业企业350家，总数达到1650家。县域服务业实现大发展，新建西柳中国商贸城、南台世界皮革皮具城、台安东北木材物流中心、岫岩万客来物流配送中心等服务业项目52个，总投资28.2亿元，实现社会消费品零售额197.5亿元，增长24.4%。县域房地产业实现大发展，引进了四川希望集团、广州碧桂园、北京望城集团、浙江祥生集团、山东红领集团等一批国内外知名企业，新上项目64个，总投资146.3亿元，开发面积达到448.2万平方米，增长2.2倍。房地产业成为县域经济新的增长点。

（五）深入推进全国文明城市创建工作，全面加强城乡建设

2009年是鞍山城乡建设投资力度最大、建设项目最多的一年。道路桥涵建设，完成投资31.5亿元，新建和改造城市道路156条。其中，新建了万水河南路、万水河北路、南环路、灵山路、园林路南延、中华路南延、盛达大道等23条城市主次干道，改造了千山中路、胜利路、中华路、人民路等22条城市主次干道，改造街巷路111条；莘英路东线隧道建成通车，站前广场、虹桥、陈家台桥等改造工程全面实施。公用设施建设，投资14亿元，新建扩建了6个220千伏变电所、9个66千伏变电所；投资7.8亿元，新建了解放路、二台子等8座大型集中供热设施，铺设和改造供暖管网237公里；投资12亿元，续建了引汤入鞍二期供水工程和大伙房水源供水工程，铺设和改造自来水管线48.3公里、排水管线113.2公里；投资1.3亿元，铺设和改造燃气管线41.8公里。城乡环境建设，实施了万水河、杨柳河、运粮河整治工程，开工建设了胜利湖、杨柳湖、东山湖、运粮湖；新建了劳动公园、解放公园等，新增城市绿地200万平方米；对站前地区及胜利路、千山中路等全面实施亮化工程，新设和改造节能路灯3791基；大力实施了城乡绿化工程，全年植树2150万株，造林14.4万亩；继续实施了千村绿化工程。小区建设，改造居民小区136个，安装小区围栏3.23万米，新建街心公园30个，新增社区广场130个。

（六）加速发展以民生为重点的社会事业，幸福全市人民

全年为城乡群众办的八个方面43件实事全部完成，人民生活进一步改善，社会事业全面发展。

就业工作不断加强。全年实现实名制就业10.9万人，城镇登记失业率下降到3.06%。建立大学毕业生职业技能培训中心、退役士兵职业技术学校、农民工培训基地和军嫂培训基地，重点解决了大学毕业生、退役士兵等就业问题。全年普通高校毕业生就业率达到90.8%。退役士兵技能培训和推荐就业均实现100%，受到中央军委充分肯定。鞍山被确定为首批国家级创建创业型城市。

社会保障不断完善。推进城镇职工社会保险应保尽保，全年新增养老、医疗、工伤、生育保险人数13.4万人，征缴社会保险基金43.7亿元。医疗保险基本实现全覆盖，新型农村合作医疗保险参合率达到99.5%，城镇居民基本医疗保险参保37万人；10万名困难企业退休职工由城镇居民基本医疗保险全部转为城镇职工基本医疗保险。社会救助制度不断完善，建立了城乡低保边缘户救助制度，严格规范低保管理，城市低保人口由上年10.5万人下降到8.1万人。保障标准进一步提高，全市离退休人员人均月增加养老金110元；新型农村合作医疗保险筹资标准每人每年由50元提高到100元；城市低保标准平均提高20%，农村低保标准平均提高35%；重点优抚对象抚恤金标准提高15%，在乡老复员军人生活补助标准提高30%。

群众住房条件继续改善。坚持保障性住房和商品房同步发展。全年建成保障性住房230万平方米，3.4万户动迁居民喜迁新居。建设了军人之家和劳模大厦。解决了1698户农村困难家庭的住房问题。全年新开工商品房面积966万平方米，增长63.2%; 竣工面积766万平方米，增长2.2倍。城市人均住房面积由22.4平方米提高到24.2平方米。

教育事业加速发展。新建了20所农村九年一贯制寄宿学校，在全省率先完成每个乡镇拥有1所九年一贯制学校目标。改造65所农村中小学校危房和20所城区薄弱中小学校。新建海城高中，7所免费高中办学条件进一步改善。占地120万平方米的鞍山职教城一期工程建成并投入使用，首批入校学生1.35万人。辽宁科技大学、鞍山师范学院办学不断取得新成果。开工建设14所公立幼儿园。

医疗卫生事业不断发展。新建鞍山精神病医院、传染病医院、结核病医院、职业病医院、骨科医院及13个高标准社区卫生服务中心，改造47所乡镇卫生院，进一步解决了群众看病难问题。防控甲型H1N1流感工作取得阶段性效果。推进医药卫生体制改革，成为全国公立医院改革试点城市。

文化体育事业进一步发展。鞍山博物馆、鞍山文化艺术大厦投入使用，科技图书馆、国际会展中心、电影大厦等文化设施加速建设；新建了12个高标准社区图书阅览室和15个农村乡镇综合文化站，在全国率先做到每个行政村都有“农家书屋”。新体育场、体育馆、游泳馆、速滑馆、乒羽馆、全民健身馆等“一场五馆”开工建设，新建了140个农村精品示范健身广场。在第十一届全运会上，鞍山籍运动员取得优异成绩，获得7.5枚金牌、9枚银牌、8枚铜牌。

群众反映的热点难点问题得到有效解决。以解决实际问题为重点，切实加强了信访工作。努力解决弃管房问题，400万平方米弃管房由政府全面接管，采取推进房改、交纳住房维修金等办法，有效解决了产权证无法办理、房屋无人管理等诸多问题。努力解决供暖问题，历年发现的供暖问题得到解决，困扰多年的铁西八家子等地区1.9万户供暖分户改造全面完成，全年新发展供暖面积1000万平方米。努力解决小区亮化问题，由政府出资，为每个社区安装了太阳能路灯，既解决了小区亮化，也减轻了居民物业费负担。努力解决群众出行问题，新增100辆城市公交车，在上年70岁以上老人免费乘车基础上，实行了60岁以上老人半价乘车。开工建设了鞍山客运北站和2个乡镇客运站，全市村通油路率100%。努力解决交通秩序问题，在主干道安装隔离护栏2.1万米，人行道安装护栏4.6万米；实施了道路交通安全专项整治行动，严厉查处交通违法违规行为，交通秩序有所改善。努力解决农民增收问题，加大政府投入，调动农民积极性，通过发展设施农业，农民人均增收800元。努力解决困难家庭子女就学问题，继续资助困难家庭高中生和大学生，为职教城学生提供生活补助。

人口与计划生育、广播电视、妇女儿童、民族、宗教、外事、侨务、对台、残联、接待、史志、档案、气象、地震、人防、民兵预备役等工作都有新成绩。

（七）推进依法行政，不断加强政府自身建设

民主法制建设不断加强。坚持依法决策和依法行政，自觉接受人大及其常委会的法律监督、工作监督和政协的民主监督，定期向人大及其常委会报告工作和向政协通报工作。认真落实人大议案，全面加强了对西鞍山的保护，全部关闭采矿选矿企业，全面治理周边环境，加强了植被恢复。对人大代表和政协委员提出的意见和建议，逐条研究，认真落实，有力地促进了政府工作和全市经济社会发展。

社会管理职能不断加强。在加强城市建设中强化社会管理，通过新建农贸市场、公共厕所等功能设施，解决马路市场和城市环境卫生问题；在加强制度建设中强化社会管理，制定和完善了《城市供热管理办法》等一系列政府规章，解决社会管理规范、公平、有序问题；在加强安全生产和消防安全中强化社会管理，持续开展安全生产和消防安全专项治理，全年排查消除各类隐患1.6万个，重特大生产安全事故为零；在平安钢都建设中强化社会管理，加强社会治安综合治理，严厉打击了各类犯罪活动，圆满完成国庆安保任务，社会保持基本稳定。

政府制度建设不断加强。完善政府决策制度，在重大资金使用、重大项目建设和重要工作安排上严格执行民主集中制，对涉及民生的重大决策实行了风险预测评估和听证制度。全面规范政府经济活动，政府采购、政府投资建设项目全部公开招标，土地出让、房产交易和产权交易全部公开挂牌，并接受全覆盖、全过程、全方位监督。全面推行政府采购项目代购制，政府投资项目代建制，较好解决了采购超标准、建设超规模、投资超预算问题。全年政府采购项目、政府投资建设项目资金节约率分别为18%和11%，鞍山做法在全省介绍经验。大力推进政务公开，保证人民群众的知情权、参与权、表达权和监督权。政府机构改革顺利完成。

政府勤政廉政和作风建设不断加强。各级政府勤奋敬业，扎实有效做好各项工作。全面加强廉政建设，严格执行机关干部廉洁自律相关规定，严厉查处了干部队伍中的违法违纪行为。全面加强软环境建设，进一步完善行政收费定值管理，规范行政审批行为，开展“服务企业百日行动”和“文明执法年”活动，鞍山成为全国投资环境百佳城市。

各位代表，过去一年，鞍山经受住金融危机的巨大冲击，取得了来之不易的发展成果，这是在市委正确领导下，市人大、市政协监督支持下，坚定不移推进结构调整、转变发展方式的结果，是坚定不移落实国家和省扩内需保增长政策措施的结果，是鞍钢等所有企业积极拼搏、战胜困难的结果，是全市上下万众一心、共同奋斗的结果。在此，我代表市人民政府向心系鞍山发展的各位代表和委员，向奋战在全市各条战线的广大干部群众，向为鞍山发展作出重大贡献的鞍钢等中省直单位和各企业，向关心和支持鞍山发展的民主党派、工商联、人民团体、驻鞍部队、武警官兵和老领导、老同志，向所有关心和参与鞍山发展的海内外朋友，表示衷心的感谢！

在肯定成绩的同时，我们也清醒地看到发展中存在的问题。主要是，调整产业结构虽然取得了一定成绩，但任务仍然十分艰巨，亟待发展与钢铁相媲美的战略性新兴产业；鞍山仍然是能源资源消耗大市，转变经济发展方式任重道远；社会不稳定因素仍然存在，一些民生问题还需要进一步解决；政府自身建设亟待加强，服务意识需要进一步提高。对于这些问题我们将在今后工作中，以更科学的办法、更有效的工作认真加以解决。

二、2010年政府工作任务

2010年是“十一五”规划的最后一年，也是“三到五年再造一个鞍山”的关键之年。新一年政府工作总体思路是，全面贯彻党的十七大、十七届三中、四中全会精神，以邓小平理论和“三个代表”重要思想为指导，深入贯彻落实科学发展观，按照市委提出的“围绕一个核心，加速两个融入，推进三大任务”总体要求，继续坚持一手抓发展、一手抓民生，一手抓城市、一手抓农村，一手抓空间、一手抓项目，实现鞍山经济社会又好又快发展。

新一年主要目标是，地区生产总值增长16%；地方财政一般预算收入增长18%；全社会固定资产投资增长30%；规模以上工业增加值增长16%；实际利用外商直接投资增长30%；引进内资增长30%；社会消费品零售总额增长20%；城市居民人均可支配收入增长10%以上，农民人均纯收入增长15%以上；单位生产总值能耗下降5.5%。全面超额完成“十一五”规划目标，为“十二五”实现新跨越奠定坚实基础。

按照上述总体思路和目标，重点做好以下六个方面工作：

（一）积极抢抓发展机遇，继续推进鞍山大发展快发展。紧紧抓住辽宁沿海经济带上升为国家战略机遇，把握世界产业转移和南资北上时机，用好用足中央继续实行积极财政政策和适度宽松货币政策，全力打造鞍山发展新高地，承担起省委、省政府提出在沈大之间实现经济隆起的重大责任。

全面推进“两带一区”建设。加快建设鞍海经济带，按照“两个融入”要求，加速打造辽宁沿海经济带和沈阳经济区互动发展区。建设腾达通海产业大道，并以腾达大道为主线，全面发展达道湾、灵山、高新区（西区）、宁远、汤岗子农高区、海城开发区、腾鳌开发区等十大工业园区；建设沈西工业走廊通海产业大道鞍山段，推进海岫和鞍台高速公路建设；建设岫岩至丹东海洋红港口一级公路，打通通海口；加快建设哈大快客、沈阳经鞍山至北京快客、岫庄铁路，努力开工建设鞍海城际轨道交通，争取规划建设凤城至岫岩、鞍山至台安铁路；力争开通鞍山至上海、广州等更多空中航线。加快建设千山文化旅游产业带，以千山东路改造和万水河、东山湖、莲花湖等“一河八湖”建设为重点，推进文化旅游产业快速发展。加快开发城市南部地区，以鞍南大道和新建国南路为中轴，加速推进基础设施建设，与老城区全面贯通；按照国家批准的土地利用规划，推进起步区建设。

继续加大招商引资力度。按照集群、企业、项目、空间“四位一体”抓招商，积极吸引央企、境外、南方民营和本地企业投资鞍山。围绕集群抓招商，工业突出五大主导产业集群招商，农业突出设施农业和特色农产品深加工集群招商，服务业突出物流大市场和精品商业招商，房地产业突出“万水千山百湖城”招商，文化旅游产业突出千山文化旅游产业带和岫岩玉皇山观光旅游产业带招商。围绕企业抓招商，按照产业集群，确定招商对象，全面引进国内外企业投资鞍山。尤其要依托鞍钢等钢铁和煤焦油资源，大力引进深加工企业。围绕项目抓招商，积极引导企业投资发展，争取全年开工建设项目2000个。围绕空间抓招商，按照产业空间布局特点，大力推进工业项目向园区集中，服务业项目向服务业集中区和精品商业带集中，房地产业项目向小区集中，设施农业项目向示范带集中，文化旅游产业项目向旅游产业带集中。

加速推进要素扩张。扩张空间要素，破解发展的空间瓶颈，为项目落地创造条件。推进城区危旧房改造，为改善民生和服务业发展创造空间；加速农村征地，拓展工业园区，为工业发展和农村城市化、城镇化创造空间；加速企业搬迁，推进向园区集中，促进企业集群化、集约化发展。扩张资本要素，破解发展的资金瓶颈，通过招商引资，吸引更多的企业资本投资鞍山。积极争取国债项目资金，大力推进银政合作和银企对接，引导企业上市融资，让更多的金融资本进入鞍山。扩张人才要素，破解发展的人才短缺瓶颈，加强蓝领人才和具有技能的白领人才培养，满足经济发展对各类人才的需求。扩张环境要素，牢固树立“政府竞争环境、企业竞争市场”理念，积极为企业发展创造良好环境，继续大力支持鞍钢等所有企业发展。

（二）加速结构调整，切实转变发展方式。坚持好中求快、快中求好、又好又快方针，进一步调整优化产业结构，全面提高经济运行质量和效益。

继续调整工业结构。全面提升传统产业。大力发展精特钢，促进鞍山由生产普碳钢为主向精特钢与普碳钢共同发展转变，重点发展取向硅钢、特种钢、高合金钢、不锈钢等精特钢产品，支持创新应用熔融还原法生产生铁，推进电炉炼钢，努力扩大精特钢生产能力。大力发展板材、线材、管材、钢结构等深加工，促进鞍山由钢铁原材料大市向钢铁精深加工大市转变，努力形成300万吨板材、200万吨线材、200万吨管材、200万吨钢结构等深加工能力，钢材深加工率达到45%以上。大力发展钢铁物流产业，促进鞍山由钢铁直销大市向钢铁物流大市转变，努力办好中国鞍山钢铁大市场，形成东北钢铁、有色等金属物流中心。大力发展装备制造及工业自动化产业，促进鞍山由钢铁生产大市向钢铁装备大市转变，重点发展矿山、焦化冶炼、轧钢以及炉外精炼和电炉等设备，装备制造占工业比重提高到20%。大力发展矿产品深加工产业，促进鞍山由矿产品原材料大市向矿产品深加工大市转变，重点发展镁合金、镁化工、镁建材、镁铸造等镁制品深加工和方解石造纸及包装印刷，矿产品深加工率提高到60%。加快发展新兴产业。加快发展以煤焦油深加工为代表的化工新材料产业集群。围绕120万吨煤焦油资源，横向到边生产蒽油、酰油等26种深加工产品，并围绕26种产品纵向到底进行系列化精深加工。利用酚油发展酚系助剂，利用萘油发展染料中间体，利用酰油发展高档有机颜料，利用蒽油发展炭黑和轮胎，利用沥青发展煤系针状焦、核石墨和炭纤维系列产品。依托中石油等石化资源和我市方解石、滑石等非金属矿产资源发展油漆涂料产品。利用鞍钢、中石油苯资源发展苯系深加工产品。利用5万吨尼龙66盐资源发展工程塑料制品。把化工新材料产业打造成千亿元产业集群。加快发展光电产业。重点发展太阳能、风能等新能源装备，电池等新动力，LED、液晶显示器等新光源，光热、地源热泵等新热源装备，促进鞍山由过去的能源消耗大市转变为新能源应用示范市和产业化基地。淘汰落后企业和产能。坚决关闭小炼钢、小水泥、小选矿、小造纸、小化工等“五小”企业。创建化工、造纸工业园区，新上化工、造纸项目全部向园区集中，区外化工、造纸企业逐步向园区搬迁。

继续调整农业结构。推进鞍山由天然农业向设施农业转变，继续建设鞍羊线、鞍营线、张庄线、腾南线设施农业示范带，全年新发展设施农业20万亩。推进鞍山农业由生产普通农产品向主要生产特色农产品转变，海城大蒜、生姜、辣根、圆葱等“四辣”农产品种植发展到6万亩，打造“四辣”基地；台安鸭鹅等家禽养殖发展到8000万只，打造鸭鹅基地；岫岩食用菌培植发展到1.5亿盘，打造蘑菇生产基地。推进鞍山农业由原材料生产向产业化方向转变，以农产品深加工园区为重点，全年新上农产品深加工项目100个，新增农业产业化龙头企业40家。推进鞍山农业由农产品找市场向市场带动农产品生产转变，建设海城耿庄农产品批发中心、台安家禽和岫岩食用菌交易市场等一批专业批发市场。推进鞍山由传统农业向创新农业转变，加强与农业院校及农业科研院所的合作，建立蘑菇、大蒜等研发中心，引进和开发农业新品种新技术，提高设施农业发展水平。

继续调整服务业结构。以城区为主体，发展现代服务业。加快流通服务业发展，推进五大现代服务业集中区建设，四达现代服务业集中区打造国内外品牌店汇聚区，中国鞍山钢铁大市场打造东北钢铁物流中心，鞍辽路五金机电大市场打造东北五金机电物流基地，解放北路汽车贸易产业带打造汽车贸易集散地，海城西柳中国商贸城打造东北小商品批发市场集中区。加快建设千山路、建国路等精品商业带，打造新华美食、韩国风情、山南中华老字号等特色商业街；规划建设一批集宾馆、酒店、写字楼、购物中心为一体的城市综合体。加快旅游服务业发展，以发展文化旅游产业为重点，做大做强千山旅游品牌；加快开发汤岗子温泉资源，大力发展健康产业；以建设中国玉博物馆为重点，打造岫岩玉皇山观光旅游产业带；加速推进牛庄老街改造；恢复和开发鞍山城、析木城，以及灵山寺、翰林院等三寺一院。加快金融服务业发展，推进鞍山市商业银行股份制改造，实现投资主体多元化，成为区域性银行；继续引进外埠商业银行，促进浦发、民生、华夏、招商和光大等商业银行建成营业；继续发展村镇银行、小额贷款公司、担保公司；加快建设金融大厦、证券大厦，发展各类银行、基金公司和投资公司，壮大五环期货等期货证券市场。加快新型服务业发展，建设创意大厦，发展传媒、软件和服务外包、建筑设计、工业设计、动漫设计等创意产业；建设中介服务大厦，发展会计、审计、律师、评估、咨询等中介服务机构；建设商会协会大厦，发展各类行业协会商会；建设会展中心，发展会展经济；建设鞍山研发分中心大厦，全面引进国内外知名研发企业；建设总部大厦，引进各类公司总部进驻。

继续提高可持续发展能力。大力推进节能减排，积极创建全国新能源应用示范市，深入开展辽河流域水污染专项整治行动，力争完成全市电厂、锅炉和烧结机脱硫项目，争取污水处理厂全面建成并投入使用。全面提升科技创新能力，积极鼓励收购国外科技型企业，引进海外研发团队；加快引进高新技术企业，支持民营科技企业发展。大力发展循环经济，推广清洗技术、修复技术和表面处理技术，发展工业再制造；利用矿渣、粉煤灰等生产新型建材，实现工业废料再生产；推进中水回用、垃圾焚烧发电等，提高资源利用效率。

（三）大力发展县域经济，加快城乡一体化进程。继续支持县域经济发展，全年投入县域发展资金13.5亿元。全面提升农业产业化、农村工业化和农村城镇化水平。

按照设施农业、加工园区、农产品深加工项目和农产品批发市场“四位一体”，加快农业产业化。继续以四条设施农业示范带为重点，按照“一县一品”方向，大力发展设施种植业、设施养殖业、设施渔业、设施林业，新发展标准化温室大棚5万栋、标准化养殖小区240个。继续完善县域农产品深加工园区基础设施，海城围绕“四辣”深加工，重点推进山东一品集团有机大蒜深加工等项目建设；台安围绕鸭鹅深加工，重点推进韩国工业园肉类提取物等项目建设；岫岩围绕食用菌深加工，重点推进鞍山东达公司食用菌罐头等项目建设。

按照园区、基础设施、主导产业集群、项目“四位一体”，加快农村工业化。继续拓展县域工业园区，完善基础设施。海城加快发展以镁合金、镁化工、镁建材、镁铸造为代表的镁制品深加工产业集群，大力培育化工塑料产业集群，重点推进中国镁业金属镁等项目建设；台安加快发展木业产业集群，大力培育农药产业集群，重点推进法国ACE公司建材及家具生产线等项目建设；岫岩加快发展特石深加工产业集群，大力培育以中草药深加工为代表的保健品产业集群。全年县域工业新上项目500个，新增规模以上工业企业300家。

按照县城、城乡结合部、中心镇和村屯整合“四位一体”，加快农村城市化和城镇化。推进县城改造，海城加快建设海西新区，以海城河为内河把老城区和西柳镇连为一体，建设大海城，加速打造60万人口城市；台安加速高速公路口与老县城全面连接，建设新台安，岫岩加速大洋河两岸开发，建设新岫岩，加速把台安和岫岩打造成30万人口中小型城市。推进近郊城市化，加快千山区达道湾、宁远、东鞍山、大孤山镇等城市化进程。推进农村城镇化，重点改造腾鳌、牛庄、黄沙、偏岭、汤岗子等城镇。

（四）以创建全国文明城市为目标，继续加强城乡建设

加强城乡基础设施建设。继续加强道路桥涵建设，建设和改造鞍南大道、建国南路、建国东路、万水河南路、万水河北路等城市道路；新建自由东街隧道、曙光路沙河桥、万水河北路跨线桥、烟草岗跨线桥，改造民生路地道桥、千山东路七号桥、杨柳河大桥等，开工建设一批过街天桥和地下通道。按照沈阳铁路客运调线的要求，加快建设快客铁路鞍山西站、海城西站；建设鞍山长客北站和西站，新建牛庄、腾鳌、高力房客运站。加强电力设施建设，新建3个220千伏变电所和8个66千伏变电所，力争新建1个550千伏变电所，规划建设岫岩石湖水库和水电站。建成引汤入鞍二期供水工程，用汤河水源置换首山水源，全面改善城市用水质量。争取完成大伙房水源供水工程。加速推进地方与鞍钢合作煤制气项目。力争开工建设国电鞍山热电联产项目。以玻璃钢沼气池为重点，发展农村新能源。

全面加强生态环境建设。实施大规模植树造林工程，创建国家森林城市。继续提高城市绿化水平，建设营城子公园、万水河带状公园、杨柳河环状公园，对117条城市主次干道绿化美化亮化，继续改造社区环境，新增城市绿地200万平方米，进一步加强玉佛山等风景区管理，继续实施城市周边矿山生态恢复工程。加强农村生态环境整治，全面实施辽河流域、浑河流域、太子河流域生态恢复和绿化工程；继续实施千村绿化工程，推进“绿色村庄”建设。继续加强水系建设，加快推进万水河、杨柳河、运粮河整治工程，开工建设丁香湖，争取建成胜利湖、杨柳湖、运粮湖、东山湖等城市景观湖。全年城区空气环境质量85%天数达到国家二级标准。

（五）继续实施民生工程，全面推进以民生为重点的社会建设。更加关心人民群众工作和生活，继续为城乡群众办实事，让全市人民生活更加幸福安康。

继续做好就业工作。制定和完善鼓励全民创业的政策措施，建设青年创业孵化园、青年创业基地和大学生、退役士兵实训基地，调动全民创业积极性，创建创业型城市。大力推进就业培训，继续办好大学毕业生职业技能培训中心、退役士兵职业技术学校、农民工培训基地，积极开展普惠制就业培训，让有劳动愿望和劳动能力的求职者实现就业。按照“发展、就业、改革、保障”八字方针，继续推进鞍钢附企改革发展。

进一步完善社会保障。建立全覆盖的保障体系，大力推进城镇职工养老保险应保尽保，积极推进农村养老保险工作；进一步拓宽城镇居民基本医疗保险覆盖面，继续提高新型农村合作医疗标准，不断改善全市社会保障水平。大力发展福利保障，分期建设总床位5000张、设施完善的养老家园，让“三无”老人免费入住公立养老院；新建市老年人活动中心，争取每个社区都有老年人活动站，继续改造农村敬老院，让全市老年人享受到更多更好的社会福利；新建市残疾人活动中心；建设无障碍设施，创建全国无障碍城市。

努力解决群众住房问题。继续建设保障性住房，大力开发商品房。推进城区棚户区和危旧房改造，进一步加快回迁房建设。提供一批廉租房，改善困难家庭住房条件。开工建设一大批动迁周转房，解决动迁群众租房难、租房贵问题。大力推进城乡结合部及大孤山等矿山周边居民区改造。继续搞好农村危房改造，解决好农村困难家庭住房问题。

大力推进教育均衡发展。实施中小学校舍安全工程，继续改造一批薄弱中小学和高中。加大对农村教育投入，全面提高农村义务教育和高中办学经费，定向免费为农村中小学培训教师，鼓励优秀大学毕业生到农村任教。建设鞍山新八中。进一步提高免费高中办学水平。以职教城建设和管理为重点，继续加强职业教育，整合职业教育资源，扩大办学规模，打造全国一流的蓝领人才基地、专业化实训基地和社会化培训基地。支持高等教育发展，积极为辽宁科技大学、鞍山师范学院和辽宁林业职业技术学院发展创造良好条件。全面加强幼儿教育，大力发展公立幼儿园，降低幼儿园收费，推进幼儿教育健康发展。

加速发展医疗卫生事业。加快建设鞍山传染病医院、结核病医院、精神病医院、职业病医院、中医院、烧伤整形医院、骨科医院、糖尿病医院、口腔医院，全面规划改造市中心医院。继续完善社区卫生服务体系。推行县级医院与中国医科大学、乡镇公立医院与市中心医院远程网上诊断，提高医疗服务水平。继续招聘医学专业大学毕业生充实乡镇卫生院、村卫生室和计划生育服务站，满足农村群众就医需求。推进医药卫生体制改革，落实国家基本药物制度，建立城乡居民健康档案，促进公共卫生服务均等化。实施公立医院改革，由政府出资对市属公立医院、乡镇卫生院和社区卫生服务中心实行专项补贴，对药品和医疗设备实行统一采购，药品实行零加价销售，切实解决群众看病贵问题。

全面发展文化体育事业。建设鞍山图书馆、冶铁博物馆、电影大厦、鞍山大剧院、妇女儿童会馆、青少年活动中心、档案方志大厦等文化设施。继续开展文化进社区、进乡镇、进工地活动，丰富广大人民群众精神文化生活。积极稳妥推进文化体制改革。加快建设新体育场、体育馆、游泳馆、速滑馆、乒羽馆、全民健身馆等“一场五馆”，在每个城区各建一个体育公园，高标准建设玉佛山健身长廊，为广大市民提供更好的运动健身场所。积极发展竞技体育，在第十一届省运会上争取优异成绩。

进一步做好双拥共建工作，为实现全国双拥模范城“五连冠”目标奠定基础。继续做好人口与计划生育、广播电视、妇女儿童、民族、宗教、外事、侨务、对台、残联、接待、史志、档案、气象、地震、人防、民兵预备役等工作。

（六）加强政府自身建设，全面提高行政能力。进一步转变政府职能,加强依法行政,建设法治、服务、责任、廉洁政府。

继续推进依法行政。严格按照法定权限和程序履行职责。自觉接受人大监督，坚持向人大及其常委会报告工作，认真落实人大及其常委会的各项决议。自觉接受政协民主监督，主动与政协民主协商。认真办理人大议案、代表建议和政协提案。积极支持民主党派和工商联工作。积极支持工会、共青团、妇联、科协等群团组织开展工作。

继续强化社会管理。加强社会保障管理，完善养老、医疗、失业、工伤、生育、低保和残疾人保障数据库，逐步实现并网管理。加强公共管理，建设覆盖全城区的公共管理监控体系，实现多网合一和资源共享，促进城市管理、社会治安、交通秩序等全面改善。加强社区建设和管理，扩大社区服务中心面积，建设一批高标准社区。完善诚信体系，打造诚信鞍山。继续加强安全生产和消防安全，深入推进安全隐患排查治理，防止重特大事故发生。加强食品药品质量安全管理，保障人民群众饮食用药安全。加强应急管理，完善应急处置预案，及时应对和妥善处置各类突发事件。加强社会治安管理，强化防控体系建设，继续推进平安钢都建设，依法打击各类犯罪活动，增强群众安全感。以维护群众合法权益、解决实际问题为重点，切实做好信访工作，保持社会稳定。

继续加强政府制度建设。强化制度用权，深度推进代购代建制，进一步规范政府经济活动。坚持以制度规范行政收费行为、行政执法行为、行政罚款行为和行政审批行为，进一步优化发展环境。全面加强国有资产监管，在地方国有企业中推行重大事项决策、外派监事会和总法律顾问等三项制度。坚持政府重大决策依法审查、听证咨询、社会公示制度，提高政府工作科学化、民主化水平。

继续加强政府勤政廉政建设。始终牢记政府的权力是人民赋予的，始终牢记全心全意为人民服务的宗旨，始终牢记以人为本的发展理念，以全面振兴鞍山老工业基地为己任，以高度负责的敬业精神推进工作，以积极进取的开拓意识加速发展，以廉洁高效的务实作风造福人民，永不自满，永不懈怠，永不停步，建设大有作为、勤廉为民的政府。

新的一年，政府还有一项重要工作，就是编制《鞍山市国民经济和社会发展第十二个五年规划纲要》。我们将按照科学性、战略性和操作性原则，深入研究，集思广益，规划好鞍山未来发展的蓝图。

各位代表，鞍山老工业基地正在全面发展、全面振兴，美好的前景已经展现在我们面前。让我们在市委领导下，在市人大、市政协监督支持下，团结带领全市人民，深入贯彻落实科学发展观，充满发展激情，抢抓振兴机遇，全面开拓创新，更加扎实工作，为实现鞍山老工业基地全面振兴新跨越而奋勇前进！
